# Searching for interpretable rules for disease mutations: a simulated annealing bump hunting strategy

**DOI:** 10.1186/1471-2105-7-417

**Published:** 2006-09-19

**Authors:** Rui Jiang, Hua Yang, Fengzhu Sun, Ting Chen

**Affiliations:** 1 Molecular and Computational Biology, University of Southern California. MCB201, 1050 Childs way, Los Angeles, CA 90089–2910, USA

## Abstract

**Background:**

Understanding how amino acid substitutions affect protein functions is critical for the study of proteins and their implications in diseases. Although methods have been developed for predicting potential effects of amino acid substitutions using sequence, three-dimensional structural, and evolutionary properties of proteins, the applications are limited by the complication of the features and the availability of protein structural information. Another limitation is that the prediction results are hard to be interpreted with physicochemical principles and biological knowledge.

**Results:**

To overcome these limitations, we proposed a novel feature set using physicochemical properties of amino acids, evolutionary profiles of proteins, and protein sequence information. We applied the support vector machine and the random forest with the feature set to experimental amino acid substitutions occurring in the *E. coli *lac repressor and the bacteriophage T4 lysozyme, as well as to annotated amino acid substitutions occurring in a wide range of human proteins. The results showed that the proposed feature set was superior to the existing ones. To explore physicochemical principles behind amino acid substitutions, we designed a simulated annealing bump hunting strategy to automatically extract interpretable rules for amino acid substitutions. We applied the strategy to annotated human amino acid substitutions and successfully extracted several rules which were either consistent with current biological knowledge or providing new insights for the understanding of amino acid substitutions. When applied to unclassified data, these rules could cover a large portion of samples, and most of the covered samples showed good agreement with predictions made by either the support vector machine or the random forest.

**Conclusion:**

The prediction methods using the proposed feature set can achieve larger AUC (the area under the ROC curve), smaller BER (the balanced error rate), and larger MCC (the Matthews' correlation coefficient) than those using the published feature sets, suggesting that our feature set is superior to the existing ones. The rules extracted by the simulated annealing bump hunting strategy have comparable coverage and accuracy but much better interpretability as those extracted by the patient rule induction method (PRIM), revealing that the strategy is more effective in inducing interpretable rules.

## Background

Variants in single bases of DNA sequences yield single nucleotide polymorphisms (SNPs), among which non-synonymous single nucleotide polymorphisms (nsSNPs) occurring in protein coding regions lead to amino acid substitutions in protein products, potentially affect protein functions, and are closely related to human inherited diseases. Hence, predicting potential effects of non-synonymous single nucleotide polymorphisms and their resulting amino acid substitutions on protein functions is of central importance in modern pathological and pharmaceutical studies [1]. Recently, increasing amounts of amino acid substitutions occurring in human proteins have been detected and collected in various databases such as the *Swiss-Prot *database [2], the *Human Gene Mutation Database *(HGMD) [3], and the *Online Mendelian Inheritance in Man*^+ ^(OMIM) database [4]. Stand alone data sets such as the unbiased laboratory mutagenesis data derived from experiments on the *E. coli *lac repressor [5,6] and the bacteriophage T4 lysozyme [7] are also available. With these data sources, the prediction is typically based on a set of features derived from the sequence and structural properties, as well as the phylogenetic information of the proteins containing the substitutions. For instance, Chasman and Adams derived sequence and structure-based features from a structural model and the phylogenetic information [8]. Sunyaev et al. analyzed amino acid substitutions on the basis of protein three-dimensional structure and multiple alignments of homologous sequences [9,10]. Ferrer-Costa et al. characterized disease-associated substitutions in terms of substitution matrix, secondary structure, accessibility, free energies of transfer from water to octanol, etc. [11,12]. Saunders and Baker created mutation models by means of a variety of structural and evolutionary features [13]. Krishnan and Westhead used the physicochemical classes of residues, sequence conservation score, secondary structure, solvent accessibility, and buried charge, etc. [14]. Ng and Henikoff utilized the sequence conservation and the BLOSUM amino acid substitution matrices [15]. With a set of features ready, the prediction is conventionally performed by making use of either the standard machine learning methods such as the decision tree [16], the support vector machines [17,18], the random forest [19], the statistical and classification models [8,9,13], or certain specifically designed methods such as the SIFT (Sorting Intolerant From Tolerant amino acid substitutions) [15].

No matter what kind of method is used, the quality of the features plays an important role in predicting the potential effects of given amino acid substitutions. To construct these features, amino acid substitutions were mapped to protein 3D structures [8-10,13]; evolutionary properties were measured from statistical models [8,9]; secondary structure and accessibility were computed from various prediction programs [11,13]; database annotations were also included [12]. However, the availability of protein or homologous proteins' structures limits the scope of the applications of these methods. In addition, most of these prediction methods are complicated and the prediction results are difficult to interpret, because a large number of complicated features are used and many of them rely heavily on other computational models. Although in some methods simple features were used with some specifically designed statistical models [15], the prediction accuracy is not as high as those methods using combined multiple features [1,8-14].

A good feature set should contain as few features as possible, while each feature should have clear physicochemical meaning and is easy to be interpreted in biological terms. To achieve these objectives, we derived a novel feature set (including a continuous form and a discrete form) based on three physicochemical properties (*molecular weight, pI value*, and *hydrophobicity scale*) of amino acids, three relative frequencies of occurrences of amino acids in the secondary structures (*helices, strands*, and *turns*) of proteins with known secondary structural information, and the evolutionary profile of the protein containing the substitution. We compared the quality of the proposed feature set with other more complicated ones by applying the decision tree [16], the support vector machine [17,18], and the random forest [19] to the experimental amino acid substitution data of the *E. coli *lac repressor [5,6] and the bacteriophage T4 lysozyme [7], as well as to a wide range of human amino acid substitutions collected in the Swiss-Prot database. The results showed that our simple yet interpretable feature set was superior to other published ones [15,14,20] in terms of the area under the receiver operating characteristic (ROC) curve (AUC), the balanced error rate (BER), and the Matthews' correlation coefficient (MCC).

Although existing machine learning methods could make predictions, they acted like "black boxes" in that they were not capable of capturing physicochemical principles behind the predictions. In many circumstances, however, these hidden principles were of more importance since they could reveal how amino acid substitutions affected protein functions and why some amino acid substitutions would result in diseases. In order to explore these principles and associate amino acid substitutions with biological knowledge, we would use rule induction methods to automatically search rules for amino acid substitutions. These rules should be (1) *interpretable*, consisting of a small set of simple features; (2) *high-quality*, with very few exceptions; and (3) *general*, capable of explaining a significant number of substitutions.

In this paper, we considered rules as sub-regions (boxes) in the feature space composed of amino acid substitutions. More specifically, the boxes were defined in terms of the feature intervals. A previous method for finding boxes in the feature space was the patient rule induction method (PRIM) [21], which searched for optimal boxes using a steepest-ascent approach and was intuitively referred to as a "bump hunting" method. When applied to our problem, the PRIM had drawbacks in that the imbalance between the numbers of data samples in different categories was not considered, and some redundant features in the boxes should be manually removed and the quality of the resulting boxes significantly decreased. To overcome these shortcomings, we incorporated a new criterion called the discrimination power to take the imbalance between the numbers of data samples in different categories into consideration, and developed a novel simulated annealing bump hunting strategy which made use of the simulated annealing method to automatically discard redundant features while extracting high quality rules. We validated this strategy using heterogenous experimental amino acid substitutions occurring in both the *E. coli *lac repressor [5,6] and the bacteriophage T4 lysozyme [7], and showed that our approach could extract rules with comparable converge and accuracy but much better interpretability as those extracted by the original PRIM method. We then applied our strategy to annotated human amino acid substitutions collected in the Swiss-Prot database and successfully identified several rules which could be interpreted using physicochemical terms and were consistent with the current biological knowledge. We further applied the induced intolerant rules to unclassified human amino acid substitution data, and the results showed that these rules could cover a large portion of data samples and most of the covered samples showed good agreement with predictions made by either the support vector machine or the random forest. Beyond the highly confident predictions, these rules more importantly revealed the physicochemical principles behind the covered amino acid substitutions and explained why these substitutions would result in diseases.

## Results

### Data sources

A large number of amino acid substitutions occurring in human proteins have been collected in the Swiss-Prot protein database [2]. In version 50.0 (released on May-30-2006), the Swiss-Prot database contained 25,994 amino acid substitution entries in 4,324 human proteins, with each substitution being annotated as "Disease", "Polymorphism", or "Unclassified". For a clear and concise presentation, we would refer to amino acid substitutions with the annotation "Disease" as intolerant ones and those with the annotation "Polymorphism" as tolerant ones. In this paper, we studied human proteins having at least 20 homologous proteins in the Pfam database [22] (version 20.0, released in May-2006), and focused on the substitutions occurring in known protein domains. In total, we collected 9, 610 intolerant substitutions, 4, 556 tolerant substitutions, and 1,487 unclassified ones in 2, 579 human proteins.

In order to validate the proposed feature set and the simulated annealing bump hunting strategy, two sets of experimental amino acid substitution data for the *E. coli *lac repressor [5,6] and the bacteriophage T4 lysozyme [7] were used. In these data sets, the effects of amino acid substitutions on the function of the corresponding protein were rated and classified to four categories. In the case of the lac repressor, the four categories were "+" (no effect), "+-" (slight effect), "-+" (larger effect), and "-" (complete absence). In the case of the T4 lysozyme, the four categories were "++" (no effect), "+" (slight effect), "+/-" (larger effect), and "-" (complete absence). Following the definition used by Chasman and Adams [8], as well as by Krishnan and Westhead [14], substitutions falling into the "no effect" category were treated as tolerant ones, and substitutions in the other categories were regarded as intolerant ones. In total, for the *E. coli *lac repressor, we collected 1,187 intolerant substitutions and 1,760 tolerant ones. For the T4 lysozyme, we collected 494 intolerant substitutions and 1,048 tolerant ones.

### Prediction of the experimental amino acid substitutions

We first show that the proposed feature set can outperform other published feature sets in the prediction of potential effects of experimental amino acid substitutions occurring in the *E. coli *lac repressor [5,6] and the T4 lysozyme [7]. We performed 10-fold cross-validation experiments using both the support vector machine and the random forest with the proposed feature set on the substitution samples, calculated the area under the ROC curve (AUC), the minimum balanced error rate (BER), and the maximum Matthews' correlation coefficient (MCC), and compared them with other published results. Detailed descriptions regarding the prediction methods and the definition of the criteria are presented in the method section.

The cross-validation results using our feature set (both the continuous form and the discrete form) are shown in Table 1. First, we can see from the table that for the experimental substitutions occurring in homogenous proteins, our feature set works well with both the support vector machine and the random forest. When working with the random forest, the discrete form of our feature set can produce an AUC of 0.944, a BER of 0.125, and a MCC of 0.741 for the experimental substitutions occurring in the *E. coli *lac repressor, suggesting that about 88% of the substitutions can be predicted accurately. When working with the support vector machine, the results are slightly worse, but the continuous form of our feature set can still predict about 85% substitutions accurately. For experimental substitutions occurring in the T4 lysozyme, we obtain similar results. Second, the results show that our feature set can also work well for experimental substitutions occurring in heterogenous proteins. When applied to the mixed samples occurring in both the *E. coli *lac repressor and the T4 lysozyme, the random forest with the discrete form of our feature set can produce an AUC of 0.927, a BER of 0.148, and a MCC of 0.693, suggesting that about 85% of the substitutions can be predicted accurately. Thirdly, we notice that in our studies, the random forest works slightly better than the support vector machine with our feature set in terms of the AUC, the BER, and the MCC.

**Table 1:** Results for predicting potential effects of experimental amino acid substitutions occurring in the *E. coli lac *repressor, the bacteriophage T4 lysozyme, and the mixed samples.

		Support vector machine			Random forest		
			
		AUC	BER	MCC	AUC	BER	MCC
Continuous	Lac repressor	0.912	0.152	0.694	0.939	0.143	0.723
	T4 lysozyme	0.897	0.177	0.614	0.907	0.167	0.640
	Mixed	0.889	0.185	0.612	0.921	0.158	0.678

Discrete	Lac repressor	0.905	0.170	0.654	**0.944**	**0.125**	**0.741**
	T4 lysozyme	0.878	0.199	0.588	**0.911**	**0.167**	**0.651**
	Mixed	0.887	0.187	0.622	**0.927**	**0.148**	**0.693**

We compared the cross-validation results using our feature set with those obtained by the SIFT (Ng and Henikoff [15]) and another published feature set (Krishnan and Westhead [14]). As a sequence homology-based method, the SIFT can achieve BERs of 33% and 34% for experimental amino acid substitutions occurring in the *E. coli *lac repressor and the T4 lysozyme, respectively. By comparison, the continuous form of our feature set can achieve corresponding BERs of 14% and 17% when working with the random forest (15% and 18% when working with the support vector machine), respectively. These results suggest that our feature set can outperform the SIFT in the prediction of potential effects of experimental amino acid substitutions occurring in homogenous proteins. The published feature set by Krishnan and Westhead [14] uses 16 features, including 13 sequence based ones (the residue identities of the original and mutated residue, the physicochemical classes of these residues (hydrophobic, polar, charged, glycine), sequence conservation score at the mutated position, molecular mass shift on mutation, and hydrophobicity difference), and 3 structure based ones (secondary structure, solvent accessibility, and buried charge). When working with the support vector machine, this feature set can achieve BERs of 27%, 29%, and 28% for experimental amino acid substitutions occurring in the *E. coli *lac repressor, the T4 lysozyme, and the mixture of them, respectively, while the continuous form of our feature set can achieve corresponding BERs of 15%, 18%, and 19%, respectively. When working with the decision tree, the published feature set can achieve BERs of 16%, 20%, and 21% for experimental amino acid substitutions occurring in the *E. coli *lac repressor, the T4 lysozyme, and the mixture of them, respectively, while the continuous form of our feature set can achieve corresponding BERs of 16%, 18%, and 19%, respectively. These results suggest that our feature set can work as good as or outperform the published feature set [14] in the prediction of potential effects of experimental amino acid substitutions occurring in both homogenous and heterogenous proteins.

### Prediction of the disease related amino acid substitutions

We performed 10-fold cross-validation experiments using both the support vector machine and the random forest with the proposed feature set on amino acid substitutions occurring in highly heterogenous human proteins and collected in the Swiss-Prot database, and compared the results with other published results (Bao and Cui [20]).

The published method [20] used a complicated feature set. For every substitution pair, they directly used two three-dimensional structural information predicted by the ENVIRONMENT program [23], one secondary structural information predicted by the STRIDE program [24], and one statistical score calculated by the SIFT program [15]. Their feature set also included another feature derived from the prediction results of these programs, and the wild-type amino acid identity. Altogether, they used six features. Five of them were three-dimensional structural or statistical ones, and needed to be calculated using other programs. Due to the limited availability of three-dimensional structural information, only a small fraction of available substitutions (3, 686 intolerant ones in 323 proteins and 532 tolerant ones in 305 proteins) in the Swiss-Prot database could be considered in their method. In contrast, our proposed feature set used only sequence information and evolutionary profiles, and did not depend on any other prediction programs. Consequently, we could predict more substitutions (9, 610 intolerant ones and 4, 556 tolerant ones) in a wider range of (2, 579) human proteins.

**Figure 1:**
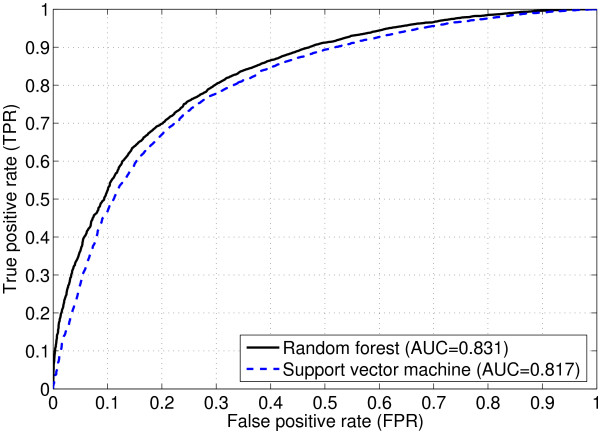
The ROC curves for predicting amino acid substitutions occurring in human proteins using the support vector machine and the random forest with the continuous form of the proposed feature set. For those using the discrete form, the curves (not shown) are similar.

For comprehensive measures, Figure 1 shows the ROC curves for the support vector machine (AUC = 0.817) and the random forest (AUC = 0.831) using the proposed continuous form of our feature set. When compared with the SIFT and the method used by Bao and Cui (both presented in [20]), we can see clearly that both methods using our feature set produce better ROC curves (see Figure 1 and Fig.1 in [20]), indicating that the proposed feature set is superior to both the SIFT and the feature set presented in [20] in terms of comprehensive prediction power (the area under the ROC curve). For the discrete form, the AUC is 0.806 for the support vector machine and 0.817 for the random forest, and the ROC curves (not shown) are similar to those using the continuous form.

More specifically, we compared the two criteria for a certain single decision threshold, as shown in Table 2. When working with the support vector machine, the continuous form of our feature set leads the SIFT (results presented in [20]) by about 4% (26% vs. 30%) in BER and about 0.15 (0.46 vs. 0.31) in MCC. When working with the random forest, the continuous form of our feature set leads the SIFT by about 5% (25% vs. 30%) in BER and about 0.18 (0.49 vs. 0.31) in MCC. Similar results are obtained when comparing the discrete form of our feature set with the SIFT. These results suggest that our feature set can outperform the SIFT in the prediction of amino acid substitutions occurring in human proteins. When comparing our results with those obtained using the feature set proposed in [20], we can see from the table that for both prediction methods using the proposed feature set, the BERs are much smaller while the MCCs are much larger than the corresponding method using the feature set presented in [20], indicating that our feature set are much better than the published one.

**Table 2:** Results for predicting potential effects of annotated amino acid substitutions occurring in human proteins and collected in the Swiss-Prot database.

	Support vector machine			Random forest		
		
	AUC	BER	MCC	AUC	BER	MCC
Bao & Cui [20]	N/A	0.318	0.274	N/A	0.292	0.315
Continuous form	0.817	0.258	0.463	**0.831**	**0.245**	**0.491**
Discrete form	0.806	0.259	0.457	0.817	0.262	0.451

### Correlation and relative importance of the proposed features

**Figure 2:**
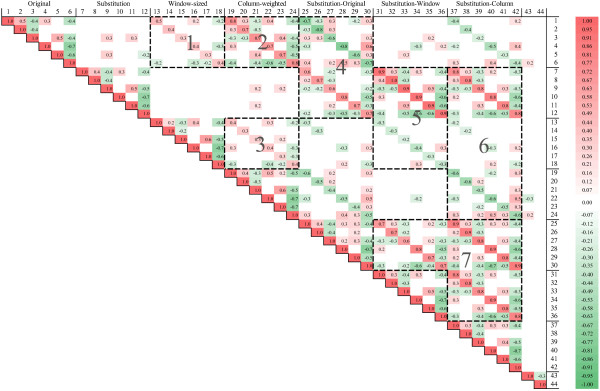
The Pearson correlation coefficient matrix (upper triangle) of the proposed features (continuous form). The Pearson correlation coefficients are calculated based on the amino acid substitutions occurring in human proteins and collected in the Swiss-Prot database. For a clear and concise presentation, correlation coefficients with absolute values less than 0.2 are ignored in the figure. For the discrete form of the proposed features, the correlation coefficient matrix (not shown) is similar.

For better understanding of the relationship between the proposed features, we calculated the pairwise Pearson's correlation coefficients between the proposed features (continuous form) based on the amino acid substitutions occurring in human proteins and presented the (upper triangle) correlation matrix in Figure 2. We divided the features to 7 groups according to their definitions in the method section, and named these groups at the top of the matrix. First, we can see from the matrix that the two evolutionary conservation scores (features 43 and 44) have very weak correlations with other 42 features. Second, for the original amino acid group (features 1 to 6), the window-sized group (features 13 to 18), and the column-weighted group (features 19 to 24), features derived from the same (physicochemical or relative frequency) properties (e.g., 1-13-19, 2-14-20, etc.) show medium positive correlations, as illustrated in region 1, 2, and 3 in the matrix. Third, the relative change features (features 25 to 42) show strong positive correlations with the substitution features derived from the same properties (e.g., 25–7, 26–8, etc.) and strong negative correlations with the original, the window-sized, or the column-weighted features (e.g., 25–1, 37–19, etc.), as illustrated in region 4, 5, and 6, respectively. Finally, as shown in region 7 in the matrix, the relative change features derived from the same properties show strong positive correlations (e.g., 25-31-37, 26-32-38, etc.). We also calculated the correlation matrix for the discrete form of the proposed features based on the amino acid substitutions occurring in human proteins and observed similar results (data not shown). These observations, though can be intuitively explained from the definitions and calculation schemes of the features (see the method section for details), provide us informative understanding and quantitative measurement of the relationship between the proposed features and can be used as evidences in the future feature selection procedure.

**Figure 3:**
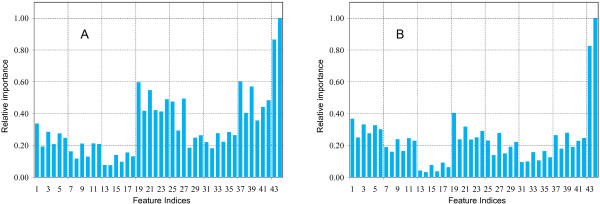
The relative importance of the proposed features. (A) the continuous form. (B) the discrete form. The raw importance for the features are calculated by the random forest [19]. The normalization is performed by dividing the raw importance with the maximum raw importance over all the features.

We then evaluated the relative importance of the proposed features using the scheme included in the random forest and presented the results in Figure 3. In the random forest, the raw importance of a feature is calculated by randomly permuting the values of the feature in the Out-Of-Bag (OOB) cases, calculating the difference of classification errors between the original and the permuted cases, and averaging this difference over all the trees in the forest [19]. To make the measurement of importance more understandable, a normalization procedure is further applied to the raw importance of each feature by dividing the raw importance with the maximum raw importance over all the features (assuming it to be a positive number). Consequently, the relative importance of every proposed feature (a real number which is less than or equal to 1.0) is obtained. For the continuous form of the proposed features (Figure 3A), we can see that the two evolutionary conservation scores (features 43 and 44) are of the most importance. The column-weighted group (features 19–24) and the substitution-column group (features 37–42) have similar importance and follow the evolutionary score group. For other groups of features, the order of importance is the substitution-original group (features 25–30) > the original group (features 1–6) > the substitution-window group (features 31–36) > the substitution group (features 7–12) > the window-sized group (features 13–18). For individual features, the first 10 most important features are ordered as *X*_44 _> *X*_43 _> *X*_37 _> *X*_19 _> *X*_39 _> *X*_21 _> *X*_27 _> *X*_24 _> *X*_42 _> *X*_25_. On the one hand, all of the 10 features except for *X*_27 _in this order are calculated with the evolutionary conservation scores (see the method section for details), revealing the significant importance of the evolutionary information in the prediction of potential effects of amino acid substitutions. On the other hand, the frequent appearances of the features derived from the molecular weight (*X*_19_, *X*_25_, and *X*_37_), the hydrophobicity scale (*X*_21_, *X*_27_, and *X*_39_), and the relative frequency in turns (*X*_24 _and *X*_42_) in this order suggest the importance of these properties in the identification of human disease related amino acid substitutions. For the discrete form of the proposed features (Figure 3B), the results show that the relative importance of the window-sized, the column-weighted, and the relative change features are not as good as their continuous forms, suggesting that the discretization procedure causes information loss for individual features. When looking at the order of the top 10 most informative features (*X*_44 _> *X*_43 _> *X*_19 _> *X*_1 _> *X*_3 _> *X*_5 _> *X*_21 _> *X*_6 _> *X*_24 _> *X*_39_), we confirm the importance of the evolutionary information (*X*_43 _and *X*_44_), the molecular weight (*X*_1 _and *X*_19_), the hydrophobicity scale (*X*_3_, *X*_21_, and *X*_39_), and the relative frequency in turns (*X*_6 _and *X*_24_).

### Validation of the simulated annealing bump hunting strategy

A merit property of the discrete form of our feature set is that every feature has strong physico-chemical meaning, which enables us to induce interpretable rules to explain the biological principles behind amino acid substitutions. We first validated the proposed simulated annealing bump hunting strategy using the heterogenous experimental amino acid substitution data. We randomly divided the mixed substitution samples occurring in the *E. coli *lac repressor and the T4 lysozyme into a training set (containing 2/3 of the data) and a test set (containing the rest 1/3 of the data), applied the simulated annealing bump hunting strategy to the training set, and evaluated the resulting rules on the test set. As an example, Table 3 lists ten rules (five intolerant ones and five tolerant ones, respectively) extracted by the simulated annealing bump hunting strategy. From the table, we can see that the extracted rules have comparable coverage (box-size), accuracy (box-mean), and discrimination power for the training and test set, suggesting that our strategy is capable of extracting general rules. For example, for intolerant rules, the simulated annealing bump hunting strategy extracted a 1-feature rule with a coverage of 0.033 and an accuracy of 0.933 from the training set, while the same rule have a coverage of 0.021 and an accuracy of 0.938 when evaluated using the test set.

**Table 3:** Results for validating the simulated annealing bump hunting strategy using the mixed experimental amino acid substitutions occurring in the *E. coli lac *repressor and the bacteriophage T4 lysozyme.

		Training set			Test set		
			
	Number of features	Box mean	Box size	Discrimination power	Box mean	Box size	Discrimination power
Intolerant	1	0.933	0.033	23.374	0.938	0.021	25.057
	2	0.934	0.020	23.794	0.917	0.015	18.383
	3	0.926	0.040	20.782	0.900	0.020	15.034
	4	0.953	0.021	33.947	0.955	0.015	35.042
	5	0.947	0.041	29.729	0.936	0.021	24.228

Tolerant	1	0.917	0.056	6.588	0.933	0.120	8.377
	2	0.955	0.052	12.734	0.944	0.048	10.168
	3	0.919	0.054	6.820	0.947	0.050	10.633
	4	0.983	0.060	35.248	0.927	0.064	7.613
	5	0.961	0.051	14.673	0.936	0.052	8.741

**Figure 4:**
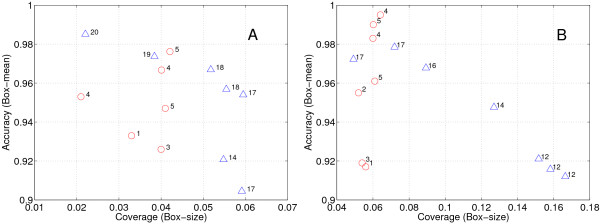
Comparison of the rules (boxes) extracted by the simulated annealing bump hunting strategy and the original bump hunting method (the SuperGEM software). (A) intolerant rules. (B) tolerant rules. Red circles are rules extracted by the proposed simulated annealing bump hunting strategy and green triangles are those extracted by the SuperGEM. The x-axes denote the coverage of the extracted rules, and the y-axes denote the accuracy of the extracted rules. The numbers beside the circles and the triangles denote the number of features included in the corresponding rules.

We also made a comparison between the simulated annealing bump hunting strategy and the original patient rule induction method (PRIM), which was implemented in the SuperGEM software [21]. Some candidate rules (7 intolerant ones and 7 tolerant ones) are shown in Figure 4. From the figure, we can see that the rules extracted by the simulated annealing bump hunting strategy have comparable coverage and accuracy but much better interpretability (less number of features) as the rules extracted by the original PRIM method. For example, for intolerant rules (Figure 4A), the simulated annealing bump hunting strategy extracted a 5-feature rule with a coverage of 0.042 and an accuracy of 0.98, while the original PRIM method extracted a 19 feature rule with comparable coverage and accuracy. Similarly, for tolerant rules (Figure 4B), the simulated annealing bump hunting strategy extracted a 4-feature rule with a coverage of 0.06 and an accuracy of 0.98, while the original PRIM method extracted a 17 feature rule with comparable coverage and accuracy.

### Amino acid substitution rules

**Figure 5:**
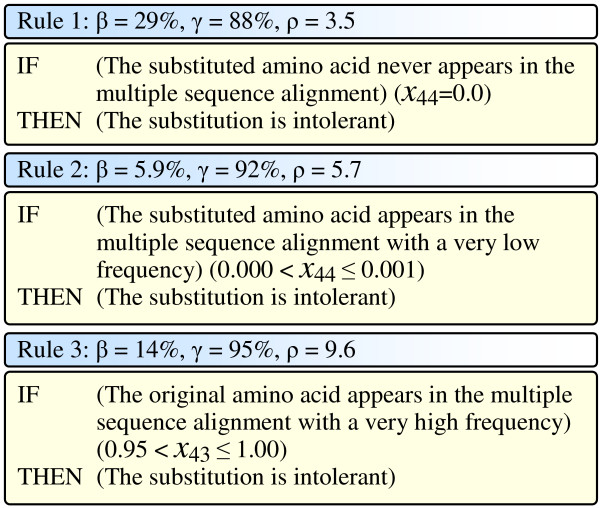
Three general intolerant rules. In the figure, *β*, *γ*, and *ρ *for each rule are the coverage, the accuracy, and the discrimination power of the corresponding rule, respectively.

We applied the simulated annealing bump hunting strategy with the discrete form of our feature set to the human amino acid substitution data and extracted several rules which were consistent with current biological knowledge. As examples, Figure 5 presents a group of three intolerant rules which uses conservation scores and provides us general understanding regarding the intolerant substitutions. Detailed descriptions regarding the notations and definitions of the features are presented in the method section.

Rule 1 in Figure 5 says that for a substitution pair, if the substituted amino acid never appears in the column (corresponding to the substitution position) of the Pfam multiple sequence alignment, the substitution is very likely to be intolerant. This rule uses a single feature (*X*_44_) and covers 29% (4,137 out of 14,166) data samples with an accuracy of 88% and a discrimination power of 3.5.

Rule 2 in Figure 5 says that for a substitution pair, if the substituted amino acid rarely (e.g., with a very low frequency ≤ 0.1%) appears in the corresponding column of the Pfam multiple sequence alignment, the substitution is very likely to be intolerant. This rule uses a single feature (*X*_44_), covering 5.9% (836) data samples with an accuracy of 92% and a discrimination power of 5.7.

Rule 3 in Figure 5 says that for a substitution pair, if the original amino acid very abundantly (with a very high frequency ≥ 0.95) appears in the corresponding column of the Pfam multiple sequence alignment, the substitution is very likely to be intolerant. This rule uses a single feature (*X*_43_), covering 14% (2, 014) data samples with an accuracy of 95% and a discrimination power of 9.6.

These rules can be understood as follows. In the Pfam multiple sequence alignments, homologous proteins are aligned according to their functional units (protein domains). Hence, amino acids appearing in a certain column of an alignment would be those that are crucial in maintaining the protein function. On the contrary, amino acids rarely appearing in a certain column of an alignment would very likely be irrelevant to the protein function. Therefore, in Rule 1 and Rule 2, when an amino acid is substituted by another amino acid which never or rarely appears in the multiple sequence alignment, the function of the protein could hardly be maintained. In Rule 3, the very abundant appearance of the original amino acid in the multiple sequence alignment indicates that the amino acid is crucial in keeping the protein function. Therefore, when the amino acid is substituted, the protein would very likely be malfunction.

**Figure 6:**
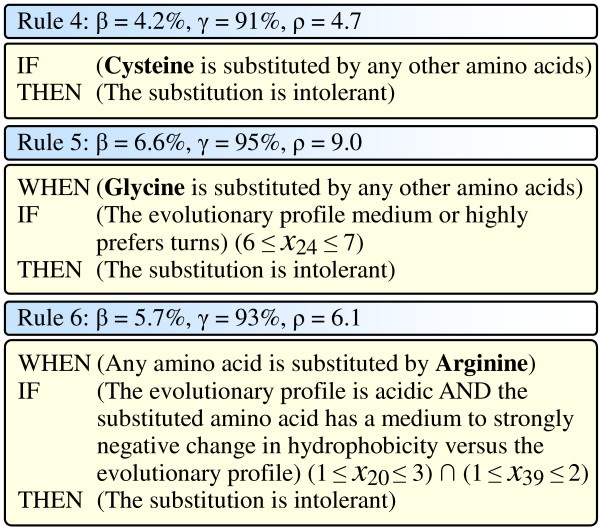
Three intolerant rules for individual amino acids. In the figure, *β*, *γ*, and *ρ *for each rule are the coverage, the accuracy, and the discrimination power of the corresponding rule, respectively.

The second group of three rules uses physicochemical features and provides us specific understanding regarding the intolerant substitutions for individual amino acids, as illustrated in Figure 6.

Rule 4 in Figure 6 says that if a Cysteine is substituted, no matter what kind of amino acids it is substituted to, the substitution is very likely to be intolerant. This rule uses a single feature (*X*_1_), covering 4.2% (596) data samples with an accuracy of 91% and a discrimination power of 4.7. The Cysteine is the only amino acid capable of forming disulfide bonds, and the disulfide bridges between Cysteines within a polypeptide support the protein's secondary structure. Therefore, when a Cysteines is substituted, the structure would be destroyed, and the protein would lose its function.

Rule 5 in Figure 6 says that when a Glycine is substituted, if the evolutionary profile medium or highly prefers turns (*x*_24 _= 6,7), the substitution is very likely to be intolerant. This rule uses 2 features (*X*_1 _and *X*_24_), covering 6.6% (940) data samples with an accuracy of 95% and a discrimination power of 9.0. This rule can be understood from two aspects. First, the Glycine is the smallest amino acid. Therefore, when a Glycine is substituted by any other (bigger) amino acids, there might not be enough space to hold that amino acid, and thus the secondary structure of the polypeptide would be destroyed. As a result, the protein would lose its function. Second, the Glycine is one of the amino acids most prefer turns (only second to Proline). Hence, when the turn structure is important to the protein function (evolutionary profile medium or highly prefers turns) and a Glycine is substituted, the function of the protein would very likely change.

Rule 6 in Figure 6 says that when an amino acid is substituted by an Arginine, if the evolutionary profile is acidic (*x*_20 _= 1,2,3), and the substituted amino acid (the Arginine) has a medium to strongly negative change in hydrophobicity versus the evolutionary profile (*x*_39 _= 1,2), the substitution is very likely to be intolerant. This rule uses 3 features (*X*_9_, *X*_20_, and *X*_39_), covering 5.7% (805) data samples with an accuracy of 93% and a discrimination power of 6.1. This rule can be understood from the following aspects. First, the Arginine is the most alkalic (with the highest pI value) and most hydrophilic amino acid. Second, an acidic evolutionary profile indicates that amino acids with small pI values are crucial to the protein's function. Thirdly, the substituted amino acid (the Arginine) having medium to strongly negative change in hydrophobicity scale suggests that hydrophilic amino acids are the majority in the homologous proteins (in other words, hydrophilic amino acids are crucial to the protein's function). Therefore, when an Arginine replaces the original amino acid, the above second and third conditions are violated, and thus the function of the protein would be destroyed.

**Figure 7:**
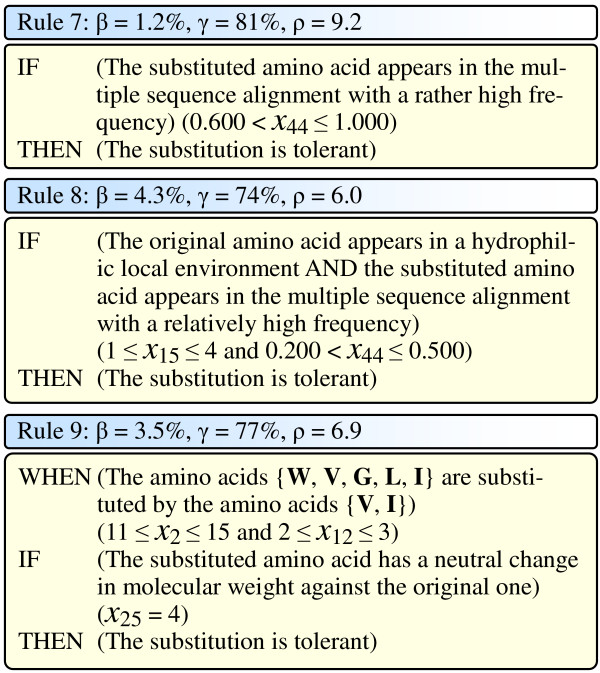
Three general tolerant rules. In the figure, *β*, *γ*, and *ρ *for each rule are the coverage, the accuracy, and the discrimination power of the corresponding rule, respectively.

The third group of three rules uses both the conservation scores and the physicochemical features, and provides us specific understanding regarding the tolerant substitutions, as illustrated in Figure 7.

Rule 7 in Figure 7 says that if the substituted amino acid appears in the multiple sequence alignment with a rather high frequency (0.600 <*X*_44 _≤ 1.000), the substitution is very likely to be tolerant. This rule uses a single feature (*X*_44_), covering 1.2% (171) data samples with an accuracy of 81% and a discrimination power of 9.2. This rule can be thought of as the opposite of the previous Rule 1 and Rule 2. Amino acids appearing in a certain column of a Pfam multiple sequence alignment would be those that are crucial in maintaining the protein function. Therefore, when an amino acid is substituted by another amino acid which appears in the multiple sequence alignment with a rather high frequency, the function of the protein could possibly be maintained, and the substitution is likely to be tolerant.

Rule 8 in Figure 7 says that if the original amino acid appears in a hydrophilic local environment (1 ≤ *X*_15 _≤ 4) and the substituted amino acid appears in the multiple sequence alignment with a relatively high frequency (0.200 <*X*_44 _≤ 0.500), the substitution is very likely to be tolerant. This rule uses 2 features (*X*_15 _and *X*_44_), covering 4.3% (610) data samples with an accuracy of 74% and a discrimination power of 6.0. The understanding of this rule is similar to the previous Rule 7. Amino acids appearing in a certain column of a Pfam multiple sequence alignment would be those that relate to the maintenance of the protein function. Hence, when an amino acid is substituted by another amino acid which appears in the multiple sequence alignment with a relatively high frequency, the function of the protein could possibly be maintained, and the substitution is likely to be tolerant.

Rule 9 in Figure 7 says that when one of the amino acids in the set of {W, V, G, L, I} is substituted by one of the amino acids the set of {V, I} (11 ≤ *X*_2 _≤ 15 and 2 ≤ *X*_12 _≤ 3), if the substituted amino acid has a neutral change in molecular weight against the original one (*X*_25 _= 4), the substitution is likely to be tolerant. This rule uses 3 features (*X*_2_, *X*_12_, and *X*_25_), covering 3.5% (493) data samples with an accuracy of 77% and a discrimination power of 6.9. The principle behind this rule is that when amino acids are substituted by other amino acids having similar physicochemical properties, the structure of the protein is likely to be maintained, and thus the function of the protein is likely to be kept.

### Prediction of the unclassified amino acid substitutions

**Figure 8:**
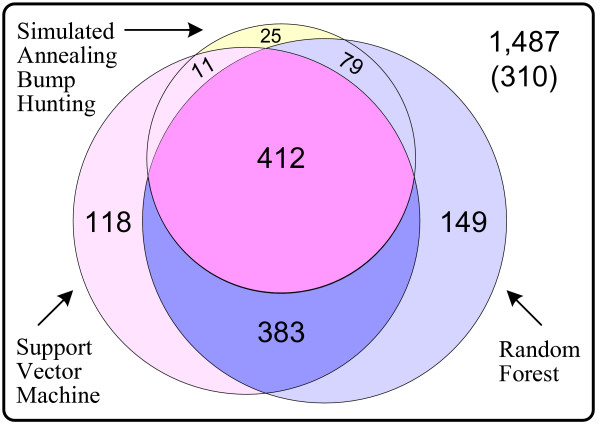
Prediction results for unclassified amino acid substitutions occurring in human proteins and collected in the Swiss-Prot database.

We further applied the support vector machine and the random forest with the discrete form of our feature set to predict potential effects of unclassified amino acid substitutions in human proteins. We first used the 10-fold cross-validation experiments to determine the decision threshold for each method so that the balanced error rate (BER) could be minimized in the experiments, and then applied each method with the corresponding decision threshold on the unclassified data to make predictions. The results are shown in Figure 8.

Within the 1,487 unclassified amino acid substitutions, the support vector machine predicted 924 (412 + 383+118+11) as intolerant and 563 (310+149 + 79 + 25) as tolerant, while the random forest predicted 1023 (412 + 383 + 149 + 79) as intolerant and 464 (310 + 118 + 11 + 25) as tolerant. 795 (412 + 383) substitutions were predicted as intolerant and 335 (310 + 25) were predicted as tolerant by both methods. These overlapping predictions were therefore with high confidence.

We also applied the six intolerant rules induced by the simulated annealing bump hunting strategy in the previous section to the unclassified data. In total, the six intolerant rules covered 527 (412 + 79 + 11 + 25) data samples, and 412 out of them were also predicted as intolerant by both the support vector machine and the random forest. Besides, 90 samples covered by these rules were also predicted as intolerant by one of the prediction methods. Only 25 samples were not predicted as intolerant by either method. These statistics suggested that the induced interpretable rules were general (covering a significant proportion of data samples), and were of very high quality (with very few exceptions). More importantly, beyond the highly confident predictions, these rules also revealed the physicochemical principles behind the covered amino acid substitutions and explained why these substitutions would be intolerant.

## Discussion and conclusions

Most contemporary studies aiming at predicting potential effects of amino acid substitutions made use of complicated and not widely available properties of amino acids and proteins. To overcome these limitations, we proposed a feature set based on three physicochemical properties of amino acids, three relative frequencies of amino acids in the secondary structures of proteins with known secondary structure information, and two evolutionary conservation scores. We applied three machine learning methods (the decision tree, the support vector machine, and the random forest) with our feature set to experimental amino acid substitutions occurring in the *E. coli *lac repressor and the bacteriophage T4 lysozyme, and showed that the methods using our feature set could achieve preferred prediction results in terms of the area under the ROC curve, the balanced error rate, and the Matthews' correlation coefficient. We further applied the support vector machine and the random forest with our feature set to a large number of amino acid substitutions occurring in highly heterogenous human proteins, and showed that our feature set could be applied to a much wider range of human proteins and the prediction methods using our feature set were superior to those using the existing more complicated feature sets.

Although existing methods could produce reasonable predictions, they were not capable of capturing physicochemical principles behind the predictions. In many situations, however, these hidden principles were of more importance because they could uncover how amino acid substitutions affect protein functions and why some substitutions would result in diseases. In order to explore these principles, we used a novel designed rule induction method called the simulated annealing bump hunting strategy to automatically extract interpretable rules for amino acid substitutions. The induced rules were either consistent with current biological knowledge or providing new insights for the understanding of the physicochemical principles behind amino acid substitutions.

One limitation of our feature set is that we currently use the Pfam multiple sequence alignment to extract evolutionary information for the query protein sequence. As a result, we are limited to deal with amino acid substitutions occurring in known protein domains. This limitation can be overcome by using some other multiple sequence alignment method such as the PSI-BLAST and ClustalW instead of the Pfam. Another limitation of our feature set is that the number of features is large, and some of them are highly correlated. Although good results have been achieved, integrating feature selection mechanisms in prediction methods could further improve the prediction performance. This demand is especially urgent when using the support vector machine as the prediction method. A third limitation is regarding how to perform fair and comprehensive comparisons between feature sets and prediction methods proposed in different literatures, especially when the training and test samples are of different sizes and from different data sources. Although this direction is not the focus in this paper, it would be of great importance and necessity in developing a general benchmark system using unified statistical criteria in our future work.

As for the simulated annealing bump hunting strategy, there exist two free parameters (*λ *and *β*_0_). Although free parameters incorporate more flexibility into the method, they make the computational burden heavier (in order to tune these parameters). How to design an automated mechanism to guide the determination of these free parameters remains an ongoing study. Also, although the nine presented rules could be well explained, there exist some other rules which are not easy to be interpreted by current biological knowledge, especially when the rules contain many features. How to simplify our feature set to make the rules more interpretable forms another research focus.

Despite the limitations, we showed that our results were reasonably good. When using our feature set with the support vector machine and the random forest, we obtained better ROC curves and smaller (balanced) prediction error rates in the cross-validation experiments. When applied to unclassified data, the six induced intolerant rules could cover a large portion of data samples, and most of the covered substitutions were also predicted as intolerant by either the support vector machine or the random forest. More importantly, beyond the highly confident predictions, these rules could also reveal the physicochemical principles behind the covered samples and explain why these substitutions would cause diseases.

## Methods

### The proposed feature set

We propose a set of 44 features which are general enough for most known proteins and are easy to be obtained by simple calculations. Our feature set has a continuous form, in which all the 44 features have continuous values, and a discrete form, in which 42 features have ordered categorical values and the other 2 have continuous values. The features are derived based on 3 physicochemical properties (*molecular weight, pI value*, and *hydrophobicity scale*) of amino acids, 3 relative frequencies for the occurrences of amino acids in the secondary structures (*helices, strands*, and *turns*) of proteins with known secondary structural information, and two evolutionary conservation scores. The unit of molecular weight is Dalton. The *p*I (Isoelectric Point) is the *p*H value at which a molecule carries no net electrical charge. The hydrophobicity scale of Kyte and Doolitle is derived from the physicochemical properties of amino acid side chains [25]. The three relative frequencies are calculated by counting the occurrences of amino acids in the corresponding secondary structure of proteins with known secondary structural information. All these six properties can either be obtained from the literature [25,26], or be calculated using only the sequential information of proteins [27].

#### The continuous form

For a given amino acid substitution pair (Org → Sub) in a certain query protein, the above 6 properties are calculated for the original (Org) and the substituted (Sub) amino acid, as well as in a window-sized situation which includes the neighbors of the original amino acids in the query protein sequence, and in a column-weighted circumstance in which the query protein sequence is aligned with its homologous proteins. The calculations of the properties for the original and the substituted amino acids are straightforward. The window-sized properties (with window size *W*) are calculated as the average of the corresponding properties for the original amino acid and its *W *- 1 neighbors in the query protein sequence. According to the known relationship between sequences and secondary structures of proteins (i.e., *α *helices are defined by repeated hydrogen bonds with a period of 4 amino acids, and have 3.6 amino acids per turn [26]), in this paper, we set the window size *W *= 9 so that the sequence information of the amino acids at the substitution positions and the *α *helices next to the substitution residues can be included. The column-weighted properties are calculated as follows. For the query protein, its homologous proteins are extracted from the Pfam database [22]. Supposing that the substitution occurs at a position corresponds to a certain column of the alignment, the column-weighted properties are then calculated as the weighted average of the corresponding properties for all the 20 kinds of amino acids, where the weight of a certain kind of amino acid is the frequency of its occurrence in the corresponding column of the alignment.

In addition, for each substitution pair, three combinations of the above four situations are considered. First, each of the 6 properties of the original amino acid is subtracted from the corresponding property of the substituted amino acid, forming 6 features measuring the relative change of the substituted amino acid versus the original amino acid. Second, each of the 6 window-sized properties is subtracted from the corresponding property of the substituted amino acid, forming 6 features measuring the relative change of the substituted amino acid versus the local environment of the substitution position in the query protein. Thirdly, each of the 6 column-weighted properties is subtracted from the corresponding property of the substituted amino acid, forming 6 features measuring the relative change of the substituted amino acid versus the evolutionary profile of the substitution position in the homologous proteins.

Besides the above physicochemical and relative frequency features, our feature set also include two evolutionary conservation scores for the original and the substituted amino acids. The conservation scores are defined as the frequencies of occurrences of the amino acids (original or substituted) in the corresponding column of the Pfam multiple sequence alignment.

With the above properties being calculated, we propose the continuous form of the feature set, including 42 physicochemical or relative frequency properties (each of the 6 amino acid properties being calculated in 7 different situations) and 2 conservation scores (for the original and the substituted amino acids). As a summary, Table 4 shows this feature set, with features labeled by *X*_*i *_for *i *= 1,..., 44.

**Table 4:** Details of the proposed features.

	Physicochemical			Relative frequency in			Conservation
	
	Molecular weight	pI value	Hydrophobicity	Helices	Strands	Turns	Frequency in MSA
Original	*X*_1_	*X*_2_	*X*_3_	*X*_4_	*X*_5_	*X*_6_	*X*_43_
Substitution	*X*_7_	*X*_8_	*X*_9_	*X*_10_	*X*_11_	*X*_l2_	*X*_44_
Window-sized	*X*_l3_	*X*_14_	*X*_15_	*X*_16_	*X*_17_	*X*_18_	
Column-weighted	*X*_19_	*X*_20_	*X*_21_	*X*_22_	*X*_23_	*X*_24_	
Substitution- Original	*X*_25_	*X*_26_	*X*_27_	*X*_28_	*X*_29_	*X*_30_	
Substitution-Window	*X*_31_	*X*_32_	*X*_33_	*X*_34_	*X*_35_	*X*_36_	
Substitution-Column	*X*_37_	*X*_38_	*X*_39_	*X*_40_	*X*_41_	*X*_42_	

Each of the 6 amino acid properties is calculated in 7 situations, forming *X*_1 _~ *X*_42_, while the conservation scores for the original and the substituted amino acids become *X*_43 _and *X*_44_, respectively.

#### The discrete form

**Figure 9:**
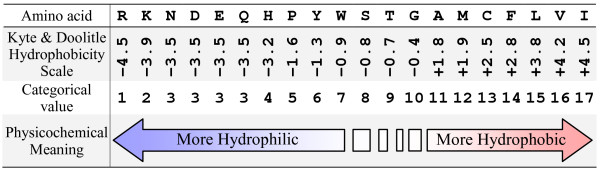
An illustration of ordered categorical values for hydrophobicity scales for the original or substituted amino acids.

In order to make the features interpretable in physicochemical terms, we further discretize the physicochemical and relative frequency properties (*X*_1 _~ *X*_42 _in Table 4). For each of the properties corresponding to the original or the substituted amino acids (*X*_1 _~ *X*_12_), we first order the possible values (corresponding to the 20 amino acids) from the smallest to the largest, and then use the ranks as the categorical values for the property. By doing this, each categorical value corresponds to one or more amino acids, while the categorical values for a certain property have intrinsic order and clear physicochemical meaning. For example, Figure 9 illustrates the ordered categorical values for the hydrophobicity scale (*X*_3 _or *X*_9_). 20 amino acids are sorted according to their hydrophobicity scale, from the most hydrophilic (R) to the most hydrophobic (I). The ranks of the sorting results are then used as the categorical values for the amino acids. Since amino acids N, D, E, and Q have identical hydrophobicity in the Kyte and Doolitle scale, they are assigned the same categorical value (3). The physicochemical meaning of the ordered categorical value is straight forward: the smaller the value, the more hydrophilic the amino acid, and vice versa.

**Figure 10:**
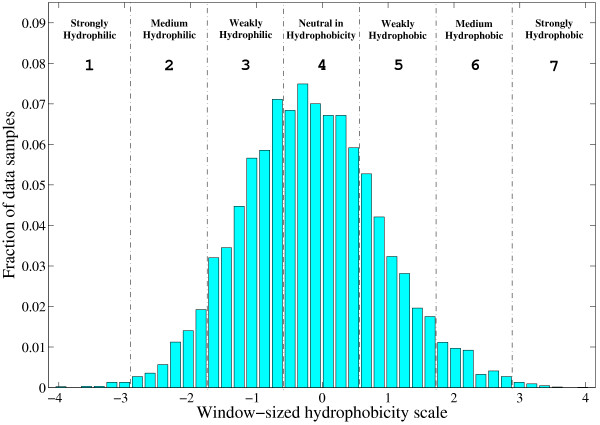
An illustration of ordered categorical values for the window-sized hydrophobicity.

For each of the other properties (*X*_13 _~ *X*_42_), we first group all the possible values to 7 bins with each bin having equal interval, and then use the indices of the bins as the categorical values of the property. By doing this, each categorical value corresponds to several substitution pairs, while the categorical values for a certain property have intrinsic order and clear physicochemical meaning. As a demonstration, Figure 10 illustrates how the window-sized hydrophobicity scale is discretized. First, all the window-sized hydrophobicity scale values (*X*_15_) in the data set are collected, and the minimum value (-4.0) and maximum value (+4.0) are determined. And then, 6 threshold values ({-2.86, -1.71, -0.57, 0.57, 1.71, 2.86}) are calculated so that the interval ([-4.0, +4.0]) can be cut into 7 equal bins. Finally, the indices of the bins are used as the categorical values. The physicochemical meaning of the ordered categorical value is straight forward: the smallest value corresponds to strongly hydrophilic local environment, while the largest value corresponds to strongly hydrophobic local environment.

With each feature having meaningful ordered categorical values, we propose the discrete form of our feature set, containing 42 physicochemical or relative frequency properties (each having ordered categorical values) and the 2 conservation scores. We would use the same method as shown in Table 4 to label these features, with *X*_1 _~ *X*_42 _having ordered categorical values.

### Prediction methods and evaluation criteria

When comparing the proposed feature set with other published ones using experimental substitution data, we use the decision tree, the support vector machine, and the random forest to predict the potential effects of amino acid substitutions. Recent studies regarding the random forest [19] have shown that prediction results can be significantly improved by growing a set of decision trees and letting them to vote. Hence, we adopt the support vector machine (SVM) [18] and the random forest (RF) [19] with the proposed feature set to predict the potential effects of human disease related substitutions. For the support vector machine, two crucial parameters are commonly referred to as C and g. We use a grid search, as included in the *libsvm *software package [18] to determine these parameters. For the random forest, two important parameters are in general referred to as mtry (the number of randomly selected features at each node of the internal decision trees) and jbt (the number of decision trees in the forest). We use jbt = 1000, and try different mtry (from 1 to 10) to select the one which can give us the best prediction performance.

The performance of each prediction method is evaluated using 10-fold cross-validation experiments, and the results of 10 independent experiments are averaged to get a fair evaluation. We use three criteria to evaluate the performance of a prediction method. The first criterion is the area under the receiver operating characteristic (ROC) curve (AUC), which provides us comprehensive understanding for the prediction power of a given method. The other two criteria are the balanced error rate (BER) and the Matthews' correlation coefficient (MCC) [28]. They take the imbalance of intolerant samples and tolerant samples into consideration and provide us more detailed understanding for the prediction power under certain decision threshold.

Given the 10-fold cross-validation results and a certain decision threshold, we can calculate the numbers of true positives (TP), true negatives (TN), false positives (FP), and false negatives (FN) under the threshold. The balanced error rate (BER) and the Matthews' correlation coefficient (MCC) [28] under the decision threshold are then defined as

BER=12(FPTN+FP+FNTP+FN),
 MathType@MTEF@5@5@+=feaafiart1ev1aaatCvAUfKttLearuWrP9MDH5MBPbIqV92AaeXatLxBI9gBaebbnrfifHhDYfgasaacH8akY=wiFfYdH8Gipec8Eeeu0xXdbba9frFj0=OqFfea0dXdd9vqai=hGuQ8kuc9pgc9s8qqaq=dirpe0xb9q8qiLsFr0=vr0=vr0dc8meaabaqaciaacaGaaeqabaqabeGadaaakeaacqqGcbGqcqqGfbqrcqqGsbGucqGH9aqpdaWcaaqaaiabigdaXaqaaiabikdaYaaadaqadiqaamaalaaabaGaeeOrayKaeeiuaafabaGaeeivaqLaeeOta4Kaey4kaSIaeeOrayKaeeiuaafaaiabgUcaRmaalaaabaGaeeOrayKaeeOta4eabaGaeeivaqLaeeiuaaLaey4kaSIaeeOrayKaeeOta4eaaaGaayjkaiaawMcaaiabcYcaSaaa@45A4@

and

MCC=TP×TN−FP×FN(TN+FN)(TN+FP)(TP+FN)(TP+FP).
 MathType@MTEF@5@5@+=feaafiart1ev1aaatCvAUfKttLearuWrP9MDH5MBPbIqV92AaeXatLxBI9gBaebbnrfifHhDYfgasaacH8akY=wiFfYdH8Gipec8Eeeu0xXdbba9frFj0=OqFfea0dXdd9vqai=hGuQ8kuc9pgc9s8qqaq=dirpe0xb9q8qiLsFr0=vr0=vr0dc8meaabaqaciaacaGaaeqabaqabeGadaaakeaacqqGnbqtcqqGdbWqcqqGdbWqcqGH9aqpdaWcaaqaaiabbsfaujabbcfaqjabgEna0kabbsfaujabb6eaojabgkHiTiabbAeagjabbcfaqjabgEna0kabbAeagjabb6eaobqaamaakaaabaGaeiikaGIaeeivaqLaeeOta4Kaey4kaSIaeeOrayKaeeOta4KaeiykaKIaeiikaGIaeeivaqLaeeOta4Kaey4kaSIaeeOrayKaeeiuaaLaeiykaKIaeiikaGIaeeivaqLaeeiuaaLaey4kaSIaeeOrayKaeeOta4KaeiykaKIaeiikaGIaeeivaqLaeeiuaaLaey4kaSIaeeOrayKaeeiuaaLaeiykaKcaleqaaaaakiabc6caUaaa@5CB9@

In general, the small the BER and the large the MCC, the better the prediction method.

### Rule induction for amino acid substitutions

With the meaningful features available, we can use rule induction methods to automatically extract interpretable rules for amino acid substitutions. A rule has a productive format

IF (*condition*)

THEN (*prediction*).

The condition part should include only a small number of features so that the rule can be easily interpreted, while the prediction part gives an assertion about the potential effects (tolerant or intolerant) of amino acid substitutions which satisfy the condition.

For a given amino acid substitution data sample, let **x **= (*x*_1_,..., *x*_*D*_)^*T *^be the vector of all the features, where *D *= 44 is the total number of features. Let *y *be the indicator of the substitution type. In the case that we target to extract rules for intolerant substitutions, *y *= 1 if a substitution is intolerant and 0, otherwise. In the case that we aim at extracting rules for tolerant substitutions, *y *has the opposite meaning. Each substitution can be thought of as an observation of the output (*y*) produced by a certain unknown function, given the inputs (**x**), and observations with similar outputs and similar inputs (or a subset of the inputs) define a rule. The similarity of the inputs can be specifically described by a "box" (sub-region) in the feature space, and defined by a set of feature intervals. The coverage of a rule can be represented by the size of the corresponding box (box-size), and the quality of a rule can be described by the average value of the output *y *for data samples inside the box (box-mean). Let *N *be the total number of data samples. The rule induction process is then mathematically formulated as:

Given repeated observations {(yk,xk)}k=1N
 MathType@MTEF@5@5@+=feaafiart1ev1aaatCvAUfKttLearuWrP9MDH5MBPbIqV92AaeXatLxBI9gBaebbnrfifHhDYfgasaacH8akY=wiFfYdH8Gipec8Eeeu0xXdbba9frFj0=OqFfea0dXdd9vqai=hGuQ8kuc9pgc9s8qqaq=dirpe0xb9q8qiLsFr0=vr0=vr0dc8meaabaqaciaacaGaaeqabaqabeGadaaakeaacqGG7bWEcqGGOaakcqWG5bqEdaWgaaWcbaGaem4AaSgabeaakiabcYcaSGqabiab=Hha4naaBaaaleaacqWGRbWAaeqaaOGaeiykaKIaeiyFa03aa0baaSqaaiabdUgaRjabg2da9iabigdaXaqaaiabd6eaobaaaaa@3D09@ composed of the substitutions (the outputs *y*_*k*_), along with simultaneous values of the features (the inputs **x**_*k*_), search in the feature space optimal boxes such that the box-means are as large as possible while the box-sizes are not very small.

This problem can be addressed using the patient rule induction method (PRIM) [21], which is also referred to as a "bump hunting" method. Each rule is described using a "box" ℬ
 MathType@MTEF@5@5@+=feaafiart1ev1aaatCvAUfKttLearuWrP9MDH5MBPbIqV92AaeXatLxBI9gBamrtHrhAL1wy0L2yHvtyaeHbnfgDOvwBHrxAJfwnaebbnrfifHhDYfgasaacH8akY=wiFfYdH8Gipec8Eeeu0xXdbba9frFj0=OqFfea0dXdd9vqai=hGuQ8kuc9pgc9s8qqaq=dirpe0xb9q8qiLsFr0=vr0=vr0dc8meaabaqaciaacaGaaeqabaWaaeGaeaaakeaaimaacqWFSeIqaaa@377E@ in the feature space, defined as

ℬ=∩d=1Dℬd,
 MathType@MTEF@5@5@+=feaafiart1ev1aaatCvAUfKttLearuWrP9MDH5MBPbIqV92AaeXatLxBI9gBamrtHrhAL1wy0L2yHvtyaeHbnfgDOvwBHrxAJfwnaebbnrfifHhDYfgasaacH8akY=wiFfYdH8Gipec8Eeeu0xXdbba9frFj0=OqFfea0dXdd9vqai=hGuQ8kuc9pgc9s8qqaq=dirpe0xb9q8qiLsFr0=vr0=vr0dc8meaabaqaciaacaGaaeqabaWaaeGaeaaakeaaimaacqWFSeIqcqGH9aqpdaafWbqaaiab=XsicnaaBaaaleaacqWGKbazaeqaaaqaaiabdsgaKjabg2da9iabigdaXaqaaiabdseaebqdcqWIPissaOGaeiilaWcaaa@4213@

where interval ℬ
 MathType@MTEF@5@5@+=feaafiart1ev1aaatCvAUfKttLearuWrP9MDH5MBPbIqV92AaeXatLxBI9gBamrtHrhAL1wy0L2yHvtyaeHbnfgDOvwBHrxAJfwnaebbnrfifHhDYfgasaacH8akY=wiFfYdH8Gipec8Eeeu0xXdbba9frFj0=OqFfea0dXdd9vqai=hGuQ8kuc9pgc9s8qqaq=dirpe0xb9q8qiLsFr0=vr0=vr0dc8meaabaqaciaacaGaaeqabaWaaeGaeaaakeaaimaacqWFSeIqaaa@377E@_*d *_= [*b*_*d*-_, *b*_*d*+_] is the boundary for the *d*-th dimension of the box. The location of the *k*-th data point in the *d*-th dimension can be described by an indicator

δkd={1,bd−≤xkd≤bd+;0,otherwise.
 MathType@MTEF@5@5@+=feaafiart1ev1aaatCvAUfKttLearuWrP9MDH5MBPbIqV92AaeXatLxBI9gBaebbnrfifHhDYfgasaacH8akY=wiFfYdH8Gipec8Eeeu0xXdbba9frFj0=OqFfea0dXdd9vqai=hGuQ8kuc9pgc9s8qqaq=dirpe0xb9q8qiLsFr0=vr0=vr0dc8meaabaqaciaacaGaaeqabaqabeGadaaakeaaiiGacqWF0oazdaqhaaWcbaGaem4AaSgabaGaemizaqgaaOGaeyypa0ZaaiqabeaafaqaaeGacaaabaGaeGymaeJaeiilaWcabaGaemOyai2aaSbaaSqaaiabdsgaKjabgkHiTaqabaGccqGHKjYOcqWG4baEdaqhaaWcbaGaem4AaSgabaGaemizaqgaaOGaeyizImQaemOyai2aaSbaaSqaaiabdsgaKjabgUcaRaqabaGccqGG7aWoaeaacqaIWaamcqGGSaalaeaacqqGVbWBcqqG0baDcqqGObaAcqqGLbqzcqqGYbGCcqqG3bWDcqqGPbqAcqqGZbWCcqqGLbqzcqGGUaGlaaaacaGL7baaaaa@54A3@

For the *k*-th data sample, another indicator is further introduced as

δk=∏d=1Dδkd,
 MathType@MTEF@5@5@+=feaafiart1ev1aaatCvAUfKttLearuWrP9MDH5MBPbIqV92AaeXatLxBI9gBaebbnrfifHhDYfgasaacH8akY=wiFfYdH8Gipec8Eeeu0xXdbba9frFj0=OqFfea0dXdd9vqai=hGuQ8kuc9pgc9s8qqaq=dirpe0xb9q8qiLsFr0=vr0=vr0dc8meaabaqaciaacaGaaeqabaqabeGadaaakeaaiiGacqWF0oazdaWgaaWcbaGaem4AaSgabeaakiabg2da9maarahabaGae8hTdq2aa0baaSqaaiabdUgaRbqaaiabdsgaKbaaaeaacqWGKbazcqGH9aqpcqaIXaqmaeaacqWGebara0Gaey4dIunakiabcYcaSaaa@3CD8@

to describe whether the *k*-th data point locates inside the box (*δ*_*k *_= 1) or not (*δ*_*k *_= 0). The size of a box ℬ
 MathType@MTEF@5@5@+=feaafiart1ev1aaatCvAUfKttLearuWrP9MDH5MBPbIqV92AaeXatLxBI9gBamrtHrhAL1wy0L2yHvtyaeHbnfgDOvwBHrxAJfwnaebbnrfifHhDYfgasaacH8akY=wiFfYdH8Gipec8Eeeu0xXdbba9frFj0=OqFfea0dXdd9vqai=hGuQ8kuc9pgc9s8qqaq=dirpe0xb9q8qiLsFr0=vr0=vr0dc8meaabaqaciaacaGaaeqabaWaaeGaeaaakeaaimaacqWFSeIqaaa@377E@ is quantified by the (normalized) number of data points falling into the box as

βℬ=1N∑k=1Nδk.
 MathType@MTEF@5@5@+=feaafiart1ev1aaatCvAUfKttLearuWrP9MDH5MBPbIqV92AaeXatLxBI9gBamrtHrhAL1wy0L2yHvtyaeHbnfgDOvwBHrxAJfwnaebbnrfifHhDYfgasaacH8akY=wiFfYdH8Gipec8Eeeu0xXdbba9frFj0=OqFfea0dXdd9vqai=hGuQ8kuc9pgc9s8qqaq=dirpe0xb9q8qiLsFr0=vr0=vr0dc8meaabaqaciaacaGaaeqabaWaaeGaeaaakeaaiiGacqWFYoGydaWgaaWcbaacdaGae4hlHieabeaakiabg2da9maalaaabaGaeGymaedabaGaemOta4eaamaaqahabaGae8hTdq2aaSbaaSqaaiabdUgaRbqabaaabaGaem4AaSMaeyypa0JaeGymaedabaGaemOta4eaniabggHiLdGccqGGUaGlaaa@4750@

The average value of the output *y *for data points locating inside the box ℬ
 MathType@MTEF@5@5@+=feaafiart1ev1aaatCvAUfKttLearuWrP9MDH5MBPbIqV92AaeXatLxBI9gBamrtHrhAL1wy0L2yHvtyaeHbnfgDOvwBHrxAJfwnaebbnrfifHhDYfgasaacH8akY=wiFfYdH8Gipec8Eeeu0xXdbba9frFj0=OqFfea0dXdd9vqai=hGuQ8kuc9pgc9s8qqaq=dirpe0xb9q8qiLsFr0=vr0=vr0dc8meaabaqaciaacaGaaeqabaWaaeGaeaaakeaaimaacqWFSeIqaaa@377E@ is referred to as the box-mean and calculated by

γℬ=1Nβℬ∑k=1Nδkyk.
 MathType@MTEF@5@5@+=feaafiart1ev1aaatCvAUfKttLearuWrP9MDH5MBPbIqV92AaeXatLxBI9gBamrtHrhAL1wy0L2yHvtyaeHbnfgDOvwBHrxAJfwnaebbnrfifHhDYfgasaacH8akY=wiFfYdH8Gipec8Eeeu0xXdbba9frFj0=OqFfea0dXdd9vqai=hGuQ8kuc9pgc9s8qqaq=dirpe0xb9q8qiLsFr0=vr0=vr0dc8meaabaqaciaacaGaaeqabaWaaeGaeaaakeaaiiGacqWFZoWzdaWgaaWcbaacdaGae4hlHieabeaakiabg2da9maalaaabaGaeGymaedabaGaemOta4Kae8NSdi2aaSbaaSqaaiab+XsicbqabaaaaOWaaabCaeaacqWF0oazdaWgaaWcbaGaem4AaSgabeaakiabdMha5naaBaaaleaacqWGRbWAaeqaaaqaaiabdUgaRjabg2da9iabigdaXaqaaiabd6eaobqdcqGHris5aOGaeiOla4caaa@4D4B@

These definitions make both the box-size and the box-mean taking values in the interval of [0, 1].

The PRIM then intends to search in the box space a box ℬ
 MathType@MTEF@5@5@+=feaafiart1ev1aaatCvAUfKttLearuWrP9MDH5MBPbIqV92AaeXatLxBI9gBamrtHrhAL1wy0L2yHvtyaeHbnfgDOvwBHrxAJfwnaebbnrfifHhDYfgasaacH8akY=wiFfYdH8Gipec8Eeeu0xXdbba9frFj0=OqFfea0dXdd9vqai=hGuQ8kuc9pgc9s8qqaq=dirpe0xb9q8qiLsFr0=vr0=vr0dc8meaabaqaciaacaGaaeqabaWaaeGaeaaakeaaimaacqWFSeIqaaa@377E@ which has maximum box-mean γℬ
 MathType@MTEF@5@5@+=feaafiart1ev1aaatCvAUfKttLearuWrP9MDH5MBPbIqV92AaeXatLxBI9gBamrtHrhAL1wy0L2yHvtyaeHbnfgDOvwBHrxAJfwnaebbnrfifHhDYfgasaacH8akY=wiFfYdH8Gipec8Eeeu0xXdbba9frFj0=OqFfea0dXdd9vqai=hGuQ8kuc9pgc9s8qqaq=dirpe0xb9q8qiLsFr0=vr0=vr0dc8meaabaqaciaacaGaaeqabaWaaeGaeaaakeaaiiGacqWFZoWzdaWgaaWcbaacdaGae4hlHieabeaaaaa@3957@, with the constraint βℬ
 MathType@MTEF@5@5@+=feaafiart1ev1aaatCvAUfKttLearuWrP9MDH5MBPbIqV92AaeXatLxBI9gBamrtHrhAL1wy0L2yHvtyaeHbnfgDOvwBHrxAJfwnaebbnrfifHhDYfgasaacH8akY=wiFfYdH8Gipec8Eeeu0xXdbba9frFj0=OqFfea0dXdd9vqai=hGuQ8kuc9pgc9s8qqaq=dirpe0xb9q8qiLsFr0=vr0=vr0dc8meaabaqaciaacaGaaeqabaWaaeGaeaaakeaaiiGacqWFYoGydaWgaaWcbaacdaGae4hlHieabeaaaaa@3951@ > *β*_0 _(*β*_0 _is a predefined threshold). This is treated by a "top-down peeling" algorithm and a "bottom-up pasting" algorithm. The top-down peeling algorithm starts from the whole search space (the initial box) and repeatedly tries to maximize the box-mean by removing some bad data points (*y *= 0) from the box. Since each peeling is performed without knowledge of later peels, it is possible that the final box can be refined by readjusting some of its boundaries. Hence, the bottom-up pasting algorithm repeatedly tries to put some good data points (*y *= 1) back by growing the box. Smaller boxes often results in larger box-mean, the PRIM thus seeks for a reasonable tradeoff between the box size and the box mean. The tradeoff is typically done manually by looking at a box-size – box-mean trajectory plot. The final box represents the extracted rule. Considering that some redundant features may exist, a tradeoff between the complexity and goodness of the rule can be further considered by trying to remove some features from the rule. This is done after the final box is obtained by looking at how box-mean changes while removing some boundaries from the box.

### Simulated annealing bump hunting strategy

The PRIM can be thought of as a steepest-ascent searching method in the box space. The final box is a (local) optimum without guarantee to be the global optimum. Also, the top-down peeling removes the data points permanently in iterations. Although some of the good data points can be put back by the bottom-up pasting, the repair to the box seems to be very limited. Moreover, it is doubtable that the process of removing some features from the rule after the final box is obtained could be an effective way to generate an optimum rule. These considerations motivate us to explore an automated feature selection methodology which can discard redundant features while extracting rules. The basic idea is to use the simulated annealing strategy instead of the steepest-ascent searching, while incorporating the automated feature selection process in the strategy.

The presence of a feature in a rule can be described as the presence of a boundary in a box and represented by an indicator *ξ*_*d*_, where *ξ*_*d *_= 1 if the *d*-th feature is included in the box and 0, otherwise. The indicator *δ*_*k *_with *ξ*_*d *_included then becomes δk=∏d=1D(δkd)ξd
MathType@MTEF@5@5@+=feaafiart1ev1aaatCvAUfKttLearuWrP9MDH5MBPbIqV92AaeXatLxBI9gBaebbnrfifHhDYfgasaacH8akY=wiFfYdH8Gipec8Eeeu0xXdbba9frFj0=OqFfea0dXdd9vqai=hGuQ8kuc9pgc9s8qqaq=dirpe0xb9q8qiLsFr0=vr0=vr0dc8meaabaqaciaacaGaaeqabaqabeGadaaakeaaiiGacqWF0oazdaWgaaWcbaGaem4AaSgabeaakiabg2da9maaradabaWaaeWaceaacqWF0oazdaqhaaWcbaGaem4AaSgabaGaemizaqgaaaGccaGLOaGaayzkaaaaleaacqWGKbazcqGH9aqpcqaIXaqmaeaacqWGebara0Gaey4dIunakmaaCaaaleqabaGae8NVdG3aaSbaaWqaaiabdsgaKbqabaaaaaaa@40C1@. The formulas for the box size βℬ
 MathType@MTEF@5@5@+=feaafiart1ev1aaatCvAUfKttLearuWrP9MDH5MBPbIqV92AaeXatLxBI9gBamrtHrhAL1wy0L2yHvtyaeHbnfgDOvwBHrxAJfwnaebbnrfifHhDYfgasaacH8akY=wiFfYdH8Gipec8Eeeu0xXdbba9frFj0=OqFfea0dXdd9vqai=hGuQ8kuc9pgc9s8qqaq=dirpe0xb9q8qiLsFr0=vr0=vr0dc8meaabaqaciaacaGaaeqabaWaaeGaeaaakeaaiiGacqWFYoGydaWgaaWcbaacdaGae4hlHieabeaaaaa@3951@ and box-mean γℬ
 MathType@MTEF@5@5@+=feaafiart1ev1aaatCvAUfKttLearuWrP9MDH5MBPbIqV92AaeXatLxBI9gBamrtHrhAL1wy0L2yHvtyaeHbnfgDOvwBHrxAJfwnaebbnrfifHhDYfgasaacH8akY=wiFfYdH8Gipec8Eeeu0xXdbba9frFj0=OqFfea0dXdd9vqai=hGuQ8kuc9pgc9s8qqaq=dirpe0xb9q8qiLsFr0=vr0=vr0dc8meaabaqaciaacaGaaeqabaWaaeGaeaaakeaaiiGacqWFZoWzdaWgaaWcbaacdaGae4hlHieabeaaaaa@3957@ remain unchanged. Considering that the proportion of data samples from different categories may be very different, we further introduce a normalized quantity

ρℬ=∑k=1Nδkyk/∑k=1Nyk∑k=1Nδk(1−yk)/∑k=1N(1−yk)=1α×γℬ1−γℬ
 MathType@MTEF@5@5@+=feaafiart1ev1aaatCvAUfKttLearuWrP9MDH5MBPbIqV92AaeXatLxBI9gBamrtHrhAL1wy0L2yHvtyaeHbnfgDOvwBHrxAJfwnaebbnrfifHhDYfgasaacH8akY=wiFfYdH8Gipec8Eeeu0xXdbba9frFj0=OqFfea0dXdd9vqai=hGuQ8kuc9pgc9s8qqaq=dirpe0xb9q8qiLsFr0=vr0=vr0dc8meaabaqaciaacaGaaeqabaWaaeGaeaaakeaaiiGacqWFbpGCdaWgaaWcbaacdaGae4hlHieabeaakiabg2da9maalaaabaWaaabmaeaacqWF0oazdaWgaaWcbaGaem4AaSgabeaakiabdMha5naaBaaaleaacqWGRbWAaeqaaaqaaiabdUgaRjabg2da9iabigdaXaqaaiabd6eaobqdcqGHris5aOGaei4la8YaaabmaeaacqWG5bqEdaWgaaWcbaGaem4AaSgabeaaaeaacqWGRbWAcqGH9aqpcqaIXaqmaeaacqWGobGta0GaeyyeIuoaaOqaamaaqadabaGae8hTdq2aaSbaaSqaaiabdUgaRbqabaaabaGaem4AaSMaeyypa0JaeGymaedabaGaemOta4eaniabggHiLdGccqGGOaakcqaIXaqmcqGHsislcqWG5bqEdaWgaaWcbaGaem4AaSgabeaakiabcMcaPiabc+caVmaaqadabaGaeiikaGIaeGymaeJaeyOeI0IaemyEaK3aaSbaaSqaaiabdUgaRbqabaGccqGGPaqkaSqaaiabdUgaRjabg2da9iabigdaXaqaaiabd6eaobqdcqGHris5aaaakiabg2da9maalaaabaGaeGymaedabaGae8xSdegaaiabgEna0oaalaaabaGae83SdC2aaSbaaSqaaiab+XsicbqabaaakeaacqaIXaqmcqGHsislcqWFZoWzdaWgaaWcbaGae4hlHieabeaaaaaaaa@7D6D@

to measure the discrimination power of a box ℬ
 MathType@MTEF@5@5@+=feaafiart1ev1aaatCvAUfKttLearuWrP9MDH5MBPbIqV92AaeXatLxBI9gBamrtHrhAL1wy0L2yHvtyaeHbnfgDOvwBHrxAJfwnaebbnrfifHhDYfgasaacH8akY=wiFfYdH8Gipec8Eeeu0xXdbba9frFj0=OqFfea0dXdd9vqai=hGuQ8kuc9pgc9s8qqaq=dirpe0xb9q8qiLsFr0=vr0=vr0dc8meaabaqaciaacaGaaeqabaWaaeGaeaaakeaaimaacqWFSeIqaaa@377E@, where α=∑k=1Nyk/∑k=1N(1−yk)
 MathType@MTEF@5@5@+=feaafiart1ev1aaatCvAUfKttLearuWrP9MDH5MBPbIqV92AaeXatLxBI9gBaebbnrfifHhDYfgasaacH8akY=wiFfYdH8Gipec8Eeeu0xXdbba9frFj0=OqFfea0dXdd9vqai=hGuQ8kuc9pgc9s8qqaq=dirpe0xb9q8qiLsFr0=vr0=vr0dc8meaabaqaciaacaGaaeqabaqabeGadaaakeaaiiGacqWFXoqycqGH9aqpdaaeWaqaaiabdMha5naaBaaaleaacqWGRbWAaeqaaaqaaiabdUgaRjabg2da9iabigdaXaqaaiabd6eaobqdcqGHris5aOGaei4la8YaaabmaeaacqGGOaakcqaIXaqmcqGHsislcqWG5bqEdaWgaaWcbaGaem4AaSgabeaakiabcMcaPaWcbaGaem4AaSMaeyypa0JaeGymaedabaGaemOta4eaniabggHiLdaaaa@46DA@ is the ratio of the positive data samples against the negative data samples. We would take the possible imbalance between the data samples into consideration and maximize the discrimination power ρℬ
 MathType@MTEF@5@5@+=feaafiart1ev1aaatCvAUfKttLearuWrP9MDH5MBPbIqV92AaeXatLxBI9gBamrtHrhAL1wy0L2yHvtyaeHbnfgDOvwBHrxAJfwnaebbnrfifHhDYfgasaacH8akY=wiFfYdH8Gipec8Eeeu0xXdbba9frFj0=OqFfea0dXdd9vqai=hGuQ8kuc9pgc9s8qqaq=dirpe0xb9q8qiLsFr0=vr0=vr0dc8meaabaqaciaacaGaaeqabaWaaeGaeaaakeaaiiGacqWFbpGCdaWgaaWcbaacdaGae4hlHieabeaaaaa@3970@ for rule induction. Nevertheless, *α *is a constant with fixed number of data samples, maximizing the discrimination power ρℬ
 MathType@MTEF@5@5@+=feaafiart1ev1aaatCvAUfKttLearuWrP9MDH5MBPbIqV92AaeXatLxBI9gBamrtHrhAL1wy0L2yHvtyaeHbnfgDOvwBHrxAJfwnaebbnrfifHhDYfgasaacH8akY=wiFfYdH8Gipec8Eeeu0xXdbba9frFj0=OqFfea0dXdd9vqai=hGuQ8kuc9pgc9s8qqaq=dirpe0xb9q8qiLsFr0=vr0=vr0dc8meaabaqaciaacaGaaeqabaWaaeGaeaaakeaaiiGacqWFbpGCdaWgaaWcbaacdaGae4hlHieabeaaaaa@3970@ is therefore equivalent to maximizing the box mean γℬ
 MathType@MTEF@5@5@+=feaafiart1ev1aaatCvAUfKttLearuWrP9MDH5MBPbIqV92AaeXatLxBI9gBamrtHrhAL1wy0L2yHvtyaeHbnfgDOvwBHrxAJfwnaebbnrfifHhDYfgasaacH8akY=wiFfYdH8Gipec8Eeeu0xXdbba9frFj0=OqFfea0dXdd9vqai=hGuQ8kuc9pgc9s8qqaq=dirpe0xb9q8qiLsFr0=vr0=vr0dc8meaabaqaciaacaGaaeqabaWaaeGaeaaakeaaiiGacqWFZoWzdaWgaaWcbaacdaGae4hlHieabeaaaaa@3957@, and vice versa.

For boxes with comparable box-sizes and box-means, we prefer boxes have fewer features. This is achieved by rewarding boxes with less features using the quantity of τℬ=exp⁡(−λ∑d=1Dξd)
 MathType@MTEF@5@5@+=feaafiart1ev1aaatCvAUfKttLearuWrP9MDH5MBPbIqV92AaeXatLxBI9gBamrtHrhAL1wy0L2yHvtyaeHbnfgDOvwBHrxAJfwnaebbnrfifHhDYfgasaacH8akY=wiFfYdH8Gipec8Eeeu0xXdbba9frFj0=OqFfea0dXdd9vqai=hGuQ8kuc9pgc9s8qqaq=dirpe0xb9q8qiLsFr0=vr0=vr0dc8meaabaqaciaacaGaaeqabaWaaeGaeaaakeaaiiGacqWFepaDdaWgaaWcbaacdaGae4hlHieabeaakiabg2da9iGbcwgaLjabcIha4jabcchaWjabcIcaOiabgkHiTiab=T7aSnaaqadabaGae8NVdG3aaSbaaSqaaiabdsgaKbqabaaabaGaemizaqMaeyypa0JaeGymaedabaGaemiraqeaniabggHiLdGccqGGPaqkaaa@4C9B@ where *λ *is a hyper-parameter. *λ *= 0 means that we do not take the number of features into consideration, while positive *λ *values give preference to less number of features. In this paper, we in general set *λ *= 1.0.

The simulated annealing strategy then intends to maximize the box-mean γℬ
 MathType@MTEF@5@5@+=feaafiart1ev1aaatCvAUfKttLearuWrP9MDH5MBPbIqV92AaeXatLxBI9gBamrtHrhAL1wy0L2yHvtyaeHbnfgDOvwBHrxAJfwnaebbnrfifHhDYfgasaacH8akY=wiFfYdH8Gipec8Eeeu0xXdbba9frFj0=OqFfea0dXdd9vqai=hGuQ8kuc9pgc9s8qqaq=dirpe0xb9q8qiLsFr0=vr0=vr0dc8meaabaqaciaacaGaaeqabaWaaeGaeaaakeaaiiGacqWFZoWzdaWgaaWcbaacdaGae4hlHieabeaaaaa@3957@ using as simple box as possible with the constraint that the box-size βℬ
 MathType@MTEF@5@5@+=feaafiart1ev1aaatCvAUfKttLearuWrP9MDH5MBPbIqV92AaeXatLxBI9gBamrtHrhAL1wy0L2yHvtyaeHbnfgDOvwBHrxAJfwnaebbnrfifHhDYfgasaacH8akY=wiFfYdH8Gipec8Eeeu0xXdbba9frFj0=OqFfea0dXdd9vqai=hGuQ8kuc9pgc9s8qqaq=dirpe0xb9q8qiLsFr0=vr0=vr0dc8meaabaqaciaacaGaaeqabaWaaeGaeaaakeaaiiGacqWFYoGydaWgaaWcbaacdaGae4hlHieabeaaaaa@3951@ > *β*_0_. We write this maximization problem as

max⁡γℬ+τℬ,s.t.βℬ>β0.
 MathType@MTEF@5@5@+=feaafiart1ev1aaatCvAUfKttLearuWrP9MDH5MBPbIqV92AaeXatLxBI9gBamrtHrhAL1wy0L2yHvtyaeHbnfgDOvwBHrxAJfwnaebbnrfifHhDYfgasaacH8akY=wiFfYdH8Gipec8Eeeu0xXdbba9frFj0=OqFfea0dXdd9vqai=hGuQ8kuc9pgc9s8qqaq=dirpe0xb9q8qiLsFr0=vr0=vr0dc8meaabaqaciaacaGaaeqabaWaaeGaeaaakeaafaqabeGacaaabaGagiyBa0MaeiyyaeMaeiiEaGhabaacciGae83SdC2aaSbaaSqaaGWaaiab+XsicbqabaGccqGHRaWkcqWFepaDdaWgaaWcbaGae4hlHieabeaakiabcYcaSaqaaiabbohaZjabc6caUiabbsha0jabc6caUaqaaiab=j7aInaaBaaaleaacqGFSeIqaeqaaOGaeyOpa4Jae8NSdi2aaSbaaSqaaiabicdaWaqabaGccqGGUaGlaaaaaa@4E97@

where *β*_0 _is a predefined threshold (minimum size of a box, e.g. *β*_0 _= 0.05). Define the energy function as *E *= 1 - (γℬ
 MathType@MTEF@5@5@+=feaafiart1ev1aaatCvAUfKttLearuWrP9MDH5MBPbIqV92AaeXatLxBI9gBamrtHrhAL1wy0L2yHvtyaeHbnfgDOvwBHrxAJfwnaebbnrfifHhDYfgasaacH8akY=wiFfYdH8Gipec8Eeeu0xXdbba9frFj0=OqFfea0dXdd9vqai=hGuQ8kuc9pgc9s8qqaq=dirpe0xb9q8qiLsFr0=vr0=vr0dc8meaabaqaciaacaGaaeqabaWaaeGaeaaakeaaiiGacqWFZoWzdaWgaaWcbaacdaGae4hlHieabeaaaaa@3957@ + τℬ
 MathType@MTEF@5@5@+=feaafiart1ev1aaatCvAUfKttLearuWrP9MDH5MBPbIqV92AaeXatLxBI9gBamrtHrhAL1wy0L2yHvtyaeHbnfgDOvwBHrxAJfwnaebbnrfifHhDYfgasaacH8akY=wiFfYdH8Gipec8Eeeu0xXdbba9frFj0=OqFfea0dXdd9vqai=hGuQ8kuc9pgc9s8qqaq=dirpe0xb9q8qiLsFr0=vr0=vr0dc8meaabaqaciaacaGaaeqabaWaaeGaeaaakeaaiiGacqWFepaDdaWgaaWcbaacdaGae4hlHieabeaaaaa@3975@)/2. The simulated annealing strategy repeatedly generates new boxes using meta-operations described below and seeking for energy decreasing. Let Δ*E *= *E*^new ^- *E*^old^. If a tentative new box can decrease the energy (Δ*E *< 0), it is accepted; otherwise (Δ*E *≥ 0), it is accepted with probability *π *= exp(-*κ*Δ*E/T*), where *κ *is a normalization constant (e.g., *κ *= 1.0) and *T *is a pseudo-temperature (with initial value 1.0). Three meta-operations are used to generate a new box from the current one.

1. **Left side peeling/pasting**. Select a *d*-th dimension at random, then update the left bound ary. For continuous values, let *b*_*d*- _← *b*_*d*- _+ *N *(0,1) × (*b*_*d*+ _- *b*_*d*-_), where *N *(0,1) is a real number sampled from a Normal distribution with mean 0 and standard derivation 1. For ordered categorical values, let *b*_*d*- _← *b*_*d*- _± 1.

2. **Right side peeling/pasting**. Select a *d*-th dimension at random, then update the right boundary. For continuous values, let *b*_*d*+ _← *b*_*d*+_*+N*(0,1) × (*b*_*d*+ _- *b*_*d*-_). For ordered categorical values, let *b*_*d*+ _← *b*_*d*+ _± 1.

3. **Feature including/excluding**. Select a *d*-th dimension at random, then update the *d*-th boundary by adding it to the box (*ξ*_*d *_← 1) or removing it from the box (*ξ*_*d *_← 0).

The simulated annealing bump hunting strategy can then be described as follows.

1. **Initialization**. Generate a box containing all the data samples.

2. **Random walk**. Execute one of the meta-operations at random on the current box, calculate

ΔE=12(γℬold+τℬold−γℬnew−τℬnew).
 MathType@MTEF@5@5@+=feaafiart1ev1aaatCvAUfKttLearuWrP9MDH5MBPbIqV92AaeXatLxBI9gBamrtHrhAL1wy0L2yHvtyaeHbnfgDOvwBHrxAJfwnaebbnrfifHhDYfgasaacH8akY=wiFfYdH8Gipec8Eeeu0xXdbba9frFj0=OqFfea0dXdd9vqai=hGuQ8kuc9pgc9s8qqaq=dirpe0xb9q8qiLsFr0=vr0=vr0dc8meaabaqaciaacaGaaeqabaWaaeGaeaaakeaacqqHuoarcqWGfbqrcqGH9aqpdaWcaaqaaiabigdaXaqaaiabikdaYaaacqGGOaakiiGacqWFZoWzdaqhaaWcbaacdaGae4hlHieabaGaee4Ba8MaeeiBaWMaeeizaqgaaOGaey4kaSIae8hXdq3aa0baaSqaaiab+Xsicbqaaiabb+gaVjabbYgaSjabbsgaKbaakiabgkHiTiab=n7aNnaaDaaaleaacqGFSeIqaeaacqqGUbGBcqqGLbqzcqqG3bWDaaGccqGHsislcqWFepaDdaqhaaWcbaGae4hlHieabaGaeeOBa4MaeeyzauMaee4DaChaaOGaeiykaKIaeiOla4caaa@5D9D@

3. **Acceptance**. If Δ*E *< 0, accept the random walk; otherwise, accept the walk with probability

Pr(accept) = exp(-*k*Δ*E/T*).

4. **Temperature decay**. Decrease *T *by power law: *T *← *T *× Δ*T*, where Δ*T *is a positive real number close to 1.0 (e.g., Δ*T *= 0.9999).

5. Repeat 2 ~ 4 until convergence.

## Authors' contributions

RJ designed the simulated annealing bump hunting strategy, performed the rule induction part and prepared the manuscript. HY designed the feature set and performed the prediction part. FS participated in the research design and helped to prepare the manuscript. TC initialized, designed and directed the research. All authors read and approved the final manuscript.
